# Deep learning based tissue analysis predicts outcome in colorectal cancer

**DOI:** 10.1038/s41598-018-21758-3

**Published:** 2018-02-21

**Authors:** Dmitrii Bychkov, Nina Linder, Riku Turkki, Stig Nordling, Panu E. Kovanen, Clare Verrill, Margarita Walliander, Mikael Lundin, Caj Haglund, Johan Lundin

**Affiliations:** 1 Institute for Molecular Medicine Finland FIMM, Helsinki Institute for Life Science HiLIFE, University of Helsinki, Helsinki, Finland; 2 Department of Women’s and Children’s Health, International Maternal and Child Health (IMCH), Uppsala University, Uppsala, Sweden; 3 Department of Pathology, Medicum, University of Helsinki, Helsinki, Finland; 4 Department of Pathology, University of Helsinki and HUSLAB, Helsinki University Hospital, Helsinki, Finland; 5 Nuffield Department of Surgical Sciences, NIHR Oxford Biomedical Research Centre, University of Oxford, Oxford, UK; 6 Department of Surgery, University of Helsinki and Helsinki University Hospital, Helsinki, Finland; 7 Research Programs Unit, Translational Cancer Biology, University of Helsinki, Helsinki, Finland; 8 Department of Public Health Sciences, Global Health/IHCAR, Karolinska Institutet, Stockholm, Sweden

## Abstract

Image-based machine learning and deep learning in particular has recently shown expert-level accuracy in medical image classification. In this study, we combine convolutional and recurrent architectures to train a deep network to predict colorectal cancer outcome based on images of tumour tissue samples. The novelty of our approach is that we directly predict patient outcome, without any intermediate tissue classification. We evaluate a set of digitized haematoxylin-eosin-stained tumour tissue microarray (TMA) samples from 420 colorectal cancer patients with clinicopathological and outcome data available. The results show that deep learning-based outcome prediction with only small tissue areas as input outperforms (hazard ratio 2.3; CI 95% 1.79–3.03; AUC 0.69) visual histological assessment performed by human experts on both TMA spot (HR 1.67; CI 95% 1.28–2.19; AUC 0.58) and whole-slide level (HR 1.65; CI 95% 1.30–2.15; AUC 0.57) in the stratification into low- and high-risk patients. Our results suggest that state-of-the-art deep learning techniques can extract more prognostic information from the tissue morphology of colorectal cancer than an experienced human observer.

## Introduction

Reincarnation of artificial neural networks in the form of deep learning^1–3^ has improved the accuracy of several pattern recognition tasks, such as classification of objects, scenes and various other entities in digital images. In a biomedical context promising results have been achieved in image-based diagnostics ranging from ophthalmology^4^ to diagnostic pathology^5^. Within digital pathology, quantification and classification of digitized tissue samples by supervised deep learning has shown good results even for tasks previously considered too challenging to be accomplished with conventional image analysis methods^6–8^.

Often, the purpose of many tasks in digital pathology, such as counting mitoses^9–11^, quantifying tumour infiltrating immune cells^12,13^, assessing the grade of tumour differentiation^14^ or characterization of specific tissue entities^15–17^ aim to ultimately predict patient outcome^18–22^. Therefore, an interesting question is whether these intermediate proxies for outcome could be bypassed and the novel machine learning techniques could be used to directly learn the prognostically relevant features in microscopy images of the tumour, without prior identification of the known tissue entities, e.g. mitoses, nuclear pleomorphism, infiltrating immune cells, tumour budding. Our hypothesis is that training a machine learning classifier by using patient outcome as the endpoint could reveal known prognostic morphologies, but also has the potential to identify previously unknown prognostic features.

Tissue images typically comprise a combination of a complex set of patterns and conventional design of an automated tissue classifier requires substantial domain expertise to plan which particular features to extract and feed into a classification algorithm. This task, known as feature engineering, is often laborious and time-consuming. Deep learning eliminates feature engineering and can learn representative features automatically and directly from the raw input examples such as images of tumour tissue obtained from cancer patients for diagnostic purposes. Learning by examples, i.e. when an algorithm is trained to predict a desired output (label) from input values is called supervised learning. Providing reliable labels for training is challenging and most of the established tissue entity labels will include errors due to the subjective nature of visual interpretation by the human observer^23,24^. In the case of prognostic stratification of cancer patients, the disease outcome i.e. if the patient has survived or died of the disease is likely to be a more reliable endpoint. Thus, mapping image data directly to patient survival information through a trained deep learning model should decrease variability and errors introduced by more subjective labelling of tissue entities.

Despite considerable effort devoted to the molecular profiling of colorectal cancer (CRC) tumours^25,26^, visual microscopic assessment of tissue morphology, i.e. typing and grading by a pathologist, together with assessment of tumour stage remains the key elements of colorectal cancer diagnostics. A number of non-vision based approaches for diagnosis and survival prediction in colorectal cancer were made using artificial neural networks^27–29^.

Here, we hypothesized that it could be possible to predict five-year disease specific survival of patients diagnosed with colorectal cancer directly from digitized images of haematoxylin and eosin (H&E) stained diagnostic tissue samples. Outcome prediction is crucial for patient stratification and disease subtyping to aid the clinical decision-making, e.g., choice of adjuvant therapy to achieve a more personalized and cost-effective treatment regimen.

Microscopic tissue assessment of CRC by a pathologist aims at describing the complex composition and architecture of the tumour. Characterisation of the cellular environment in which the tumour exists is as important for disease subtyping and prognostic stratification as for characterisation of the tumour itself ^30^. Both the tumour and its microenvironment is assessed according to multiple parameters including tumour budding, presence of infiltrating immune cells, lymph- and blood-vessel invasion, vascularization, tumour necrosis, glandular formation, and level of cell differentiation^31,32^.

In this study, we train a deep learning-based classifier to predict five-year disease-specific survival in a comprehensive series of digitized tumour tissue samples of CRC stained for basic morphology only. For training and testing the algorithm we used sections of tumour tissue cores (1 mm in diameter) assembled into tumour tissue microarrays (TMAs). The classifier combined two types of artificial neural networks (convolutional and recurrent) which is compared with the prognostic accuracy achieved by the visual assessment, i.e. tumour grading performed by a skilled pathologist. Our analysis suggests that even a small tissue area obtained from the primary tumour contains prognostic information that can be extracted by the use of artificial intelligence-based methods.

## Results

### Image processing and analysis pipeline

We applied a machine learning based approach for automated analysis of digitized microscopic images of CRC samples with the goal to improve prognostic stratification of patients. To this end, we obtained images of H&E stained TMA spots from 420 patients diagnosed with CRC^33^. Follow-up time and outcome information was available for each of the patients as well as clinicopathological characteristics of the tissue samples (Table 1).

**Table 1:** Patient characteristics.

Clinicopathological variable	Selected Patients	Original Set
**Patient count**	420	641
**Age at diagnosis**		
<50 years	53 (12.6%)	77 (12%)
50–64 years	123 (29.3%)	189 (29.5%)
65–74 years	145 (34.5%)	216 (33.7%)
*>=*75	99 (23.6%)	159 (24.8%)
average	65.4	65.9
**Gender**		
Female	193 (46%)	301 (47%)
Male	227 (54%)	340 (53%)
**Tumour location**		
Colon	234 (55.7%)	352 (54.9%)
Rectum	186 (44.3%)	289 (45.1%)
**Dukes’ stage**		
A	51 (12.1%)	93 (14.5%)
B	141 (33.6%)	231 (36%)
C	114 (27.1%)	166 (25.9%)
D	114 (27.1%)	149 (23.2%)
NA	0 (0%)	2 (0.3%)
**Histological grade on whole-slide**		
Low (1–2)	285 (67.9%)	439 (68.4%)
High (3–4)	135 (32.1%)	200 (32.5%)
NA	0 (0%)	2 (0.3%)
**Visual Risk Score on TMA spots**		
Low Risk	185 (44.%)	—
High Risk	191 (45.5%)	—
NA	44 (10.5%)	—
**Digital Risk Score (CNN + LSTM)**		
Low Risk	210 (50.0%)	—
High Risk	210 (50.0%)	—

**Figure 1:**
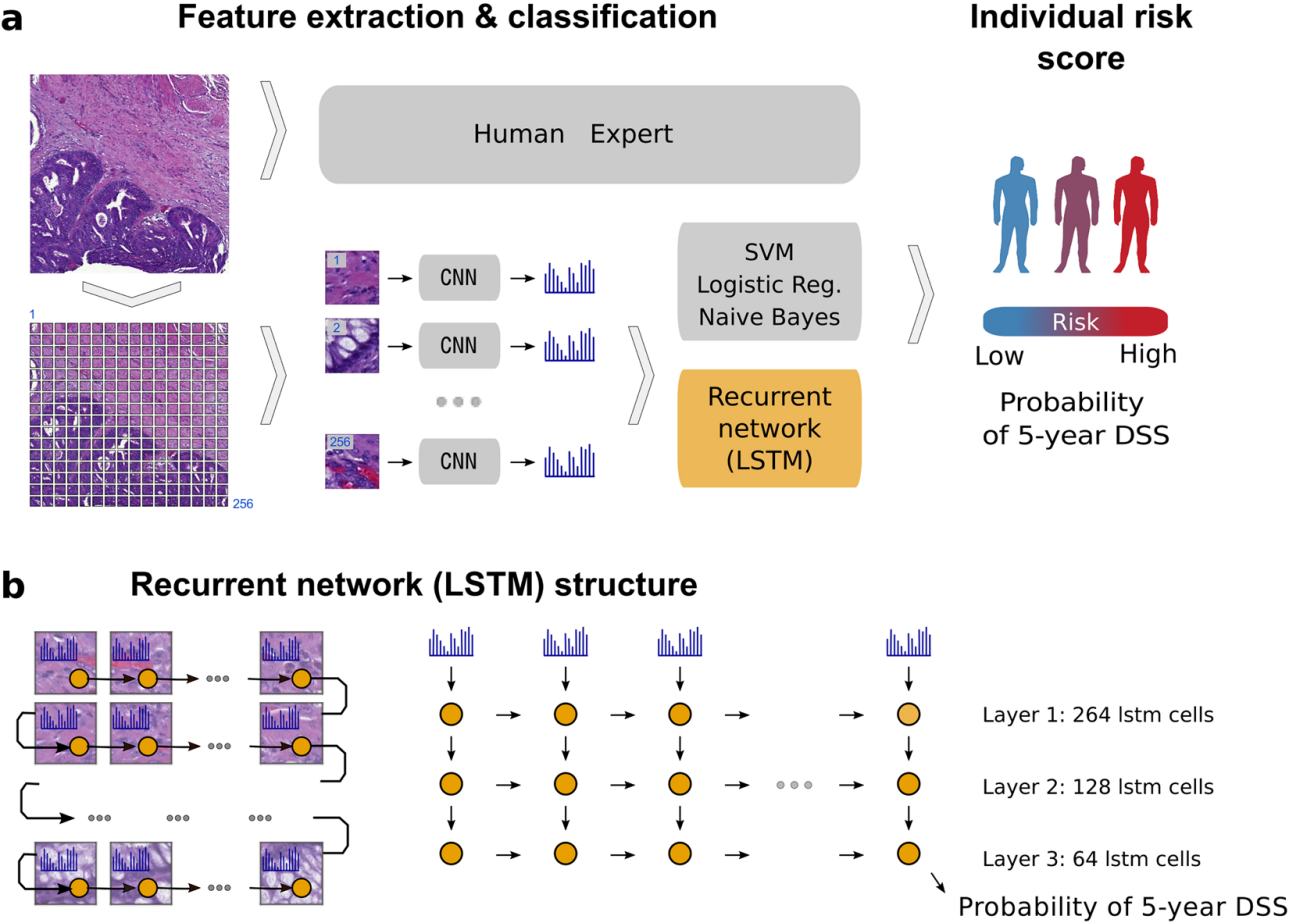
Overview of the image analysis pipeline and Long Short-Term Memory (LSTM) prognostic model. Images of tissue microarray (TMA) spots are characterized by a pre-trained convolutional neural network (VGG-16) in a tile-wise manner. The VGG-16 network produces a high-dimensional feature vector for each individual tile from an input image. These features then serve as inputs for classifiers trained to predict five-year disease-specific survival (DSS) (**A**). The Long Short-Term Memory (LSTM) Network slides through the entire image of the tissue microarray spot to jointly summarize observed image tiles and predict the patient risk score (**B**).

The image analysis workflow takes an RGB image of one TMA spot per patient as an input and first splits it into tiles (Fig. 1a). Then, a convolutional neural network extracts a high-dimensional feature vector from each tile. Here we took advantage of the VGG-16^34^ convolutional network pre-trained on the ImageNet dataset^35^. Reusing neural network trained on one domain (such as classifying objects on natural images) for similar purposes in other domains is known as transfer learning^36,37^. Finally, a recurrent neural network (Long Short-Term Memory; LSTM^38^) reads-in a sequence of VGG-16 features tile-by-tile to predict five-year disease specific survival (Fig. 1b). In case of one-dimensional (1D) LSTM architecture, tiles can be arranged in a sequence in different order. We evaluated model performance using different tile ordering including row-wise, column-wise as well as random ordering within each TMA spot image. We observed no significant difference in the model performance depending on tile order (Supplementary Fig. 1). We also tested whether two-dimensional (2D) LSTM, i.e. grid-like architecture yields performance improvement. Again, no significant difference in model performance was observed as compared to a much simpler 1D LSTM on our dataset. Overall, the recurrent nature of the LSTM architecture allows to process an arbitrary number of image tiles. Thus, it is not bound to a specific image size and enables analysis of variable-sized images, keeping the model complexity (number of free parameters) fixed.

The performance of the LSTM model was compared to conventional machine learning classifiers, i.e. Logistic Regression, Naïve Bayes and Support Vector Machine in prediction of five-year disease-specific survival. Finally, prognostic accuracy of the automated analysis was compared against visual assessment performed by skilled pathologists. Particularly, we used histological grade assessed on whole-slide tissue section, a Visual Risk Score assessed on the TMA spots by three experienced pathologists and Dukes’ stage groups as a reference. The Visual Risk Score was formed by taking a majority vote of the three experts and allowed for direct comparison of machine learning based approach with its human counterpart, as both were assessed from the TMA spot images only.

### Survival Analysis

**Figure 2:**
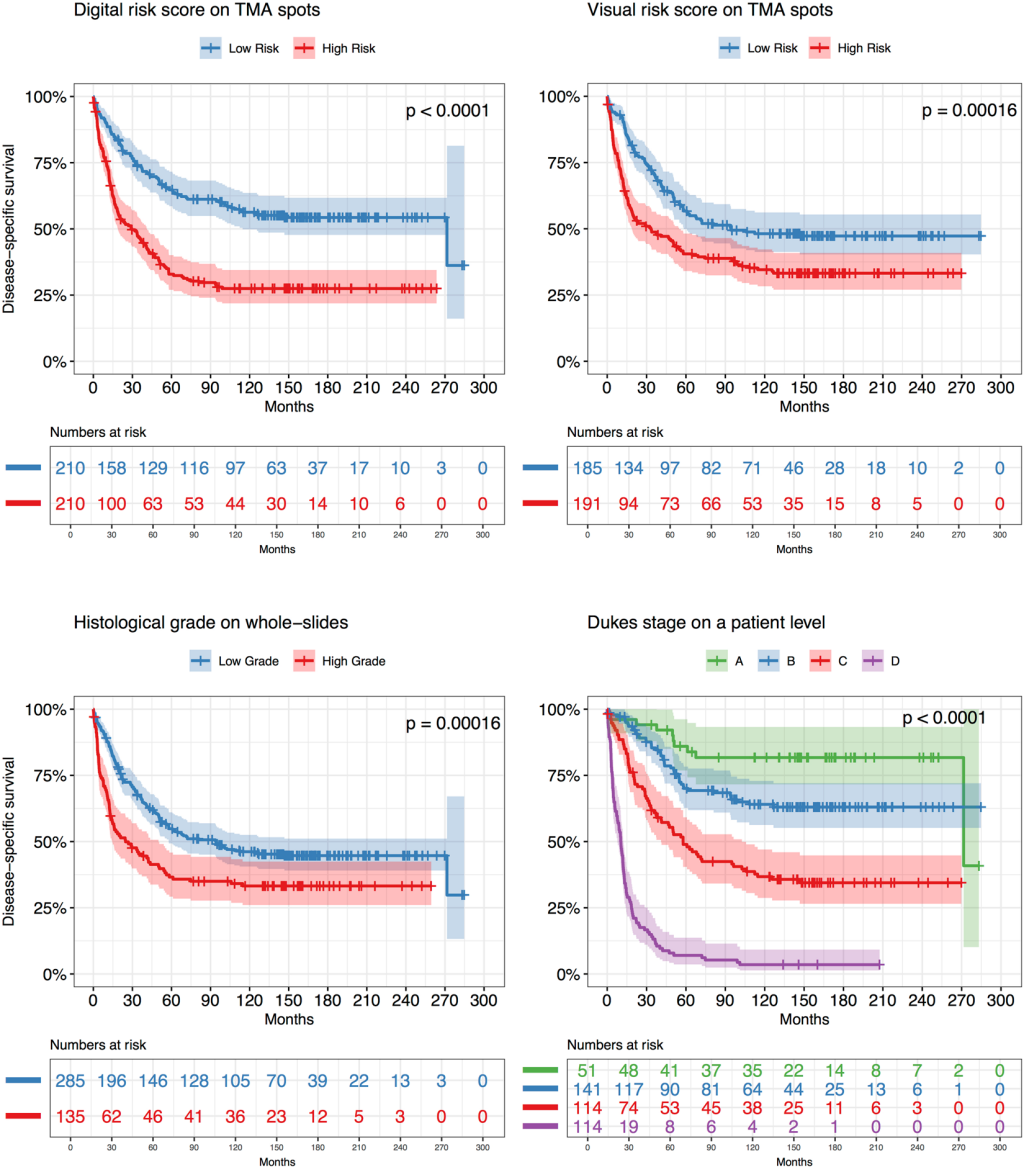
Kaplan-Meier survival curves based on different predictors for 420 colorectal cancer patients. We dichotomized patients into Low Risk group (blue curve) and High Risk group (Red curve) by median value of each predictor independently. The LSTM model predictor yields stronger stratification with a Hazard Ratio (HR) of 2.3 (log rank p-value < 0.0001) as compared to Visual Scoring on a tissue microarray (TMA) spot level (HR 1.67; log rank p-value = 0.00016) and histological grade on a whole-slide level (HR 1.65; log rank p-value = 0.00016). Dukes’ stage also stratifies the patients into groups with significantly different outcome (p-value < 0.0001). Dukes’ stage remains a stronger predictor of survival, but is not directly comparable to the tissue-based variables, since it includes extent of local invasion, number of lymph nodes affected and whether distant metastasis is observed.

**Figure 3:**
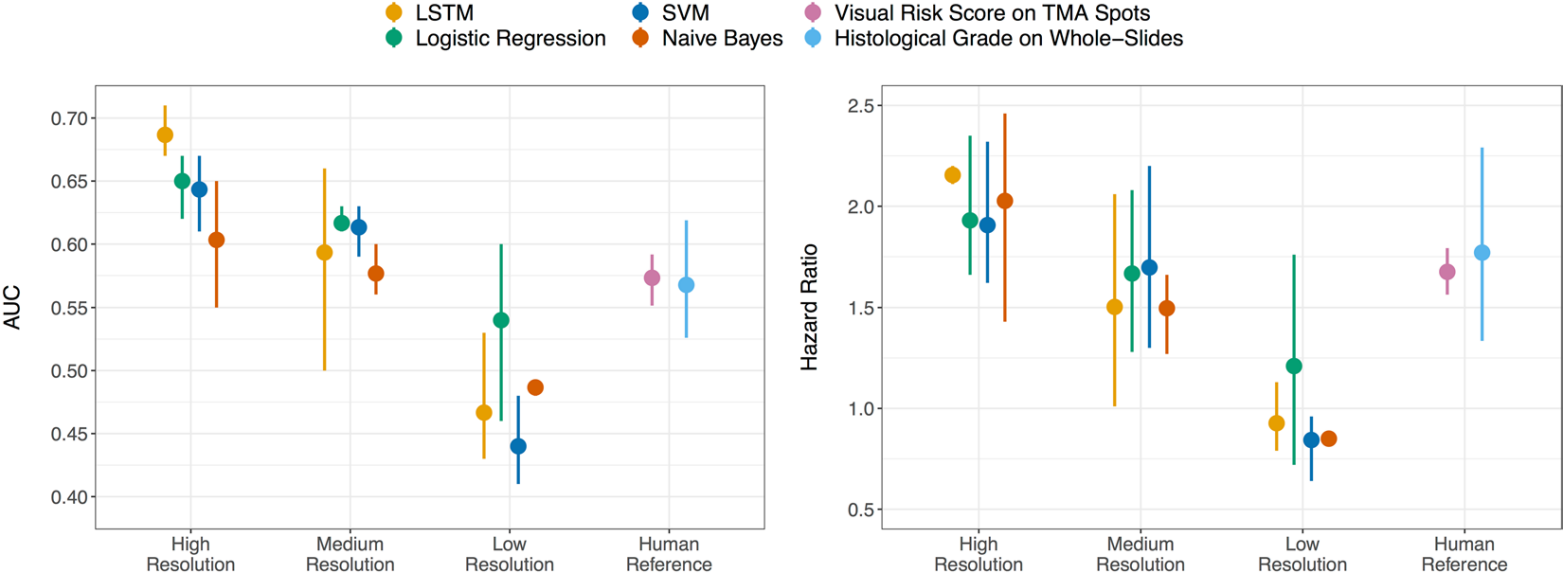
Predictive performance of four classifiers evaluated in cross-validation on images of tissue microarray (TMA) spots across different image resolutions. We trained four (Naïve Bayes, Support Vector Machine (SVM), Logistic Regression and Long Short-Term Memory Network) classifiers to predict five-year disease specific survival from tissue microarray (TMA) spot images of three different resolutions. “High” indicates images of original pixel size (0.22 μm). “Medium” and “Low” correspond to images downscaled by a factor of 4 and 16 respectively. The graphs show that high resolution images give the best performance of prognostic models as measured by area under the receiver operating characteristic curve (AUC, graph to the left) and hazard ratio (HR, graph to the right) in 3-fold cross-validation. The error bars indicate variation among folds with a dot representing the average value. Human reference corresponds both to a visual risk score assessed on a TMA level and histological grade assessed on the whole tumour sample (i.e. whole-slide) level as part of the primary diagnosis.

To compare the prognostic accuracy of our machine learning based approach to that of established clinical predictors and visual assessments we first computed survival curves using the Kaplan-Meier method. Patients were stratified into low- and high-risk groups based on the following predictors individually: the LSTM model, histological grade and the Visual Risk Score. We additionally included Dukes’ stage as a predictor with four groups (Fig. 2). We observed that the LSTM model predicts disease-specific survival with higher accuracy (average AUC: 0.69) than histological grade (AUC: 0.57) and the Visual Risk Score (AUC: 0.58) (Fig. 3). The LSTM model identified a low-risk group with 65% five-year survival compared to 33% in the high-risk group. According to histological grade assessed on the whole slide as part of the primary diagnosis, the five-year disease-specific survival in the low-risk group was 55% and in the high-risk group 36%. The Visual Risk Score was close to histological grade with 56% in low-risk group and 38% among those at high risk. The survival curves are very low due to the enrichment of events in the dataset. In order to ensure generalization of our model to independent test set, we evaluated the model on 181 patients that were not included in cross-validation. We observed that performance of the model on the held out 181 patients is comparable to that in cross-validation (Supplementary Fig. 2). We also observed that model predictions are consistent across different patient subgroups (Supplementary Fig. 3).

The AUC of the LSTM model in prediction of five-year disease-specific outcome was significantly higher compared to that of histological grade (Venkatraman’s p-value 0.003) and Visual Risk Score (Venkatraman’s p-value 0.025). Taken together, the LSTM model was significantly better in discriminating low-risk from high-risk patients with regards to colorectal cancer outcome (log-rank p-value < 0.001). We also estimated survival probabilities for Duke stages and observed that stage of disease was the strongest predictor of disease-specific survival among all four evaluated predictors (AUC 0.81). The ROC curve of Dukes’ differed significantly from other predictors (Venkatraman’s p-value < 0.001).

To estimate the prognostic effect of LSTM model score adjusted for all the other studied covariates we applied a non-parametric Cox proportional hazards (PH) model (Table 2). Univariate Cox PH regression confirmed that the LSTM model (HR: 2.3, CI 95% 1.79–3.03), histological grade (HR 1.65, CI 95% 1.30–2.15), Visual Risk Score (HR 1.67, CI 95% 1.28–2.19) and Dukes’ stage (HR 20.29, CI 95% 10.44–39.44) were all statistically significant predictors (Wald p-value < 0.001). Age at diagnosis was also identified as a significant predictor (HR 1.42 at 65 years cut-off, CI 95% 0.93–2.19). Gender was not predictive of survival and was excluded from multivariate analysis. We observed that histological grade violated the assumption of proportional hazards as the global chi-square test was significant (p-value = 0.014). Thus, the final multivariate model was stratified by histological grade. According to the model, the LSTM model score was a significant predictor (Wald p-value < 0.001) with a HR of 1.89 (CI 95% 1.41–2.53), which was slightly lower than in the univariate model due to the adjustment for the other covariates (Table 2). These results indicate that the LSTM model is an independent predictor of five-year colorectal cancer-specific survival.

**Table 2:** Univariate and Multivariate Cox proportional hazards model based on disease-specific survival in 420 patients with colorectal cancer.

Factor	Univariate			P-value	Multivariate	
	P-value (Wald)	HR	(95% CI)		HR	(95% CI)
**Age at diagnosis**	0.013					
<50 years		1			1	
50–64 years		0.98	(0.63–1.54)	0.46	1.21	(0.73–1.99)
65–74 years		1.42	(0.93–2.19)	0.008	1.91	(1.18–3.08)
75		1.64	(1.04–2.58)	<0.001	3.41	(2.06–5.65)
**Gender**	0.55					
Female		1				
Male		1.08	(0.84–1.40)			
**Dukes’ stage**	<0.001					
A		1			1	
B		2.05	(1.03–4.06)	0.23	1.65	(0.73–3.71)
C		4.78	(2.45–9.34)	<0.001	4.91	(2.24–10.78)
D		20.29	(10.44–39.44)	<0.001	21.49	(9.72–47.53)
**Histological grade on whole-slide**	<0.001					
Low (1–2)		1				
High (3–4)		1.65	(1.30–2.15)			
**Visual Risk Score on TMA spots**	<0.001			0.03		
Low Risk		1			1	
High Risk		1.67	(1.28–2.19)		1.42	(1.04–1.94)
**Digital Risk Score (CNN + LSTM)**	<0.001			<0.001		
Low Risk		1			1	
High Risk		2.3	(1.79–3.03)		1.89	(1.41–2.53)

### Model Performance Comparison

We compared the performance of the LSTM model to traditional classifiers including Naïve Bayes, Logistic regression and Support Vector Machines. All classifiers in our workflow were trained to predict the binary outcome of five-year disease-specific outcome. In a three-fold cross validation the patients were split into balanced folds with 280 patients used for training, 60 for validation and 140 held-out for testing in each fold. Comparison of the performance of the classifiers was based on the AUCs and hazard ratios as estimated on the test set: the LSTM – hazard ratio 2.3, AUC 0.69; SVM – hazard ratio 2.06, AUC 0.64; Logistic Regression – hazard ratio 1.84, AUC 0.65, Naïve Bayes – hazard ratio 1.85, AUC 0.61. Figure 3 summarizes the performance of the classifiers across individual folds. We also used different input image resolutions but kept tile size fixed to 224 by 224 pixels. This allowed us to identify optimal pixel-size for VGG-16 features. The results showed that original resolution of 0.22 μ/pixel was required to achieve the best performance for all the classifiers when using VGG-16 features (Fig. 3). We also observed that the LSTM model outperformed other classifiers according to both AUC and hazard ratios. More rigorous comparison of the models is required to conclude that predictive accuracy of the LSTM is significantly better than other classifiers.

### Network Activations Distinguish Tissue Morphologies

**Figure 4:**
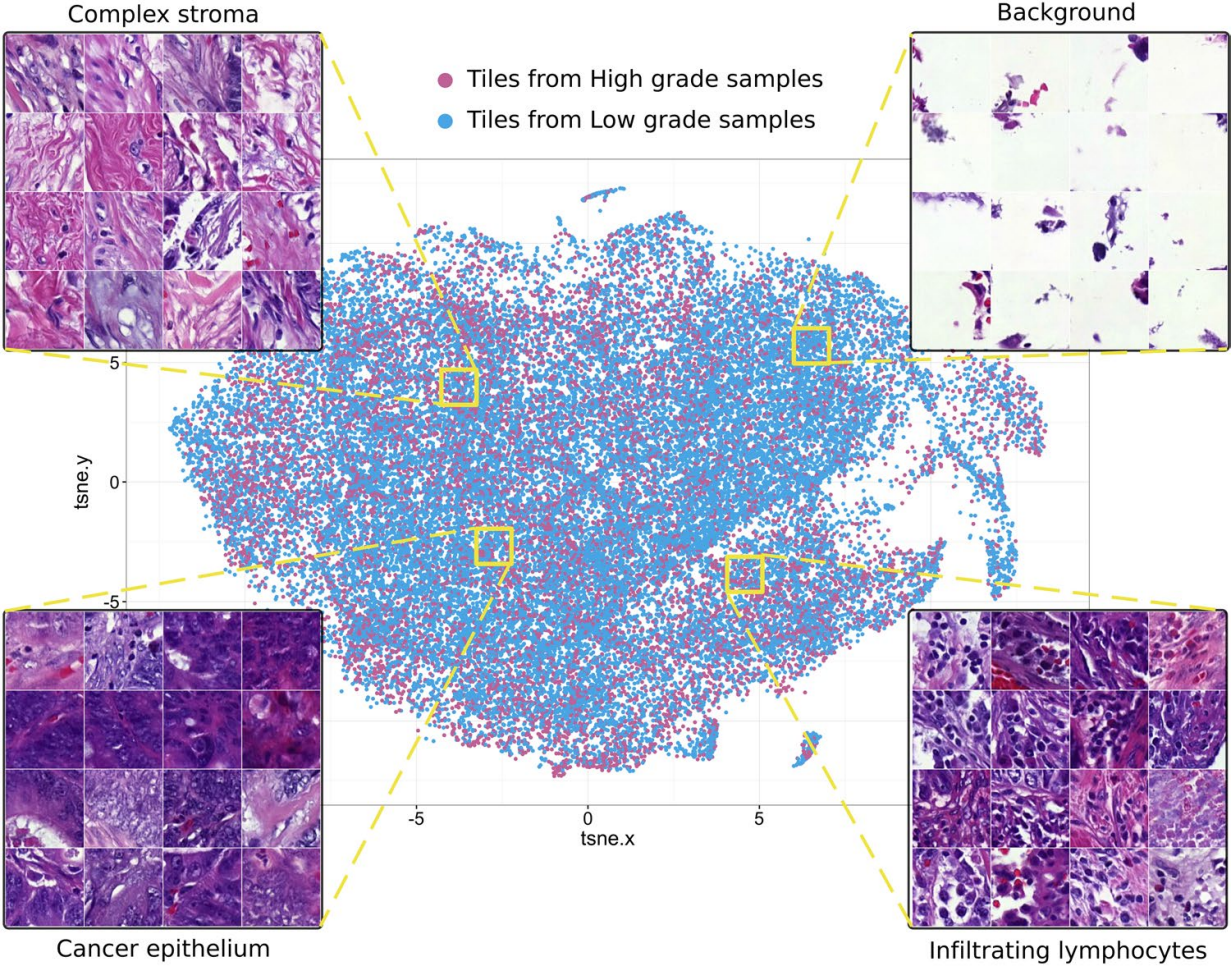
Features pre-trained on the ImageNet database distinguish tissue types in images of colorectal tumours stained for basic morphology (haematoxylin and eosin). We first extracted high-dimensional features from tissue microarray spot images split into individual tiles (n = 380,000) with the convolutional neural network model (VGG-16). Then we projected the features onto 2D plane with t-distributed Stochastic Neighbour Embedding such that each dot on the scatterplot corresponds to a tile. Each tile contains a (relatively) homogeneous tissue pattern and hence the tiles group together on the scatter plot according to pattern similarity. Zooming into local areas of the scatter plot identifies tissue entities that group together: stroma, cancer epithelium, infiltrating immune cells. This observation suggests that the VGG-16 model appears as an efficient descriptor of microscopic images of colorectal samples. Finally, tiles (indicated as points) on the scatter plot are coloured based on the histological grade of the sample they belong to.

As the VGG-16 network was used for feature extraction without additional fine-tuning, we had to ensure that extracted features were meaningful when applied to microscopic images. To evaluate this the entire image dataset was split into 380,000 tiles sized 224 × 224 pixels and processed by VGG-16 network to obtain 4096-bins-long feature vector for each tile. Then we projected the high dimensional representation of tiles onto a 2D plane with t-distributed stochastic neighbour embedding method^39^. This allowed us to check whether image tiles group together based on tissue-pattern similarity, i.e. according to morphological entities (Fig. 4). This observation suggested that VGG-16 features are also suitable for stratification of complex tissue patterns present in haematoxylin-eosin stained CRC samples. Thus, intermediate activations extracted from the VGG-16 network pre-trained on a large-scale dataset of regular photos can generalize to a broader range of problems in different domains including tissue-based pathology.

After the VGG-16 features had been extracted from each tile of the TMA spot, they were processed with a 3-layer recurrent neural network, specifically an LSTM network. The LSTM solves a classification task by “looking” at TMA spot image tiles one-by-one to predict five-year survival. Individual units, also called memory cells within the LSTM network are capable to detect and memorize tiles that contain relevant morphological entities and to disregard irrelevant tiles, such as those that contain white background. This is done through explicit gating mechanisms called input gate, forget gate and output gate. Here we made an attempt to find units that perform biologically interpretable discrimination of tissue patterns on the level of TMA spot image tiles. We hypothesized that if the observed tile contains information regarding disease outcome (e.g. poorly differentiated epithelial cells) certain memory cells within the network were expected to learn these patterns, aggregate them in the memory, and propagate them throughout the network to contribute to the final prediction.

**Figure 5:**
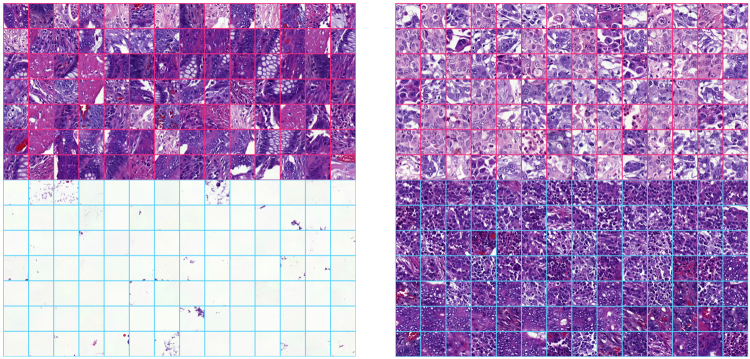
Individual units of the Long Short-Term Memory prognostic model learnt to separate tissue patterns. Tiles that correspond to extreme activations (either strong positive or strong negative) of individual units within the prognostic Long Short-Term Memory outcome prediction network are visualized. The upper half of each grid indicates the top 98 tiles with extreme positive response of a neuron and the bottom half indicates 98 most negative responses of the same neuron.

We examined gate activations and hidden activation of the memory cells as our network processed test set images. Analysis revealed several memory cells that responded to certain morphological entities, such as mucosal glands, immune cell conglomerates, and cancer epithelium (Fig. 5). Most of the meaningful activations occurred within the input gate and hidden activation of the cells within the neural network model. We could not identify any obvious activation patterns of forget gate and the majority of memory cells were not directly interpretable.

## Discussion

In this work, we investigated whether a deep learning algorithm that takes images of small regions of tumour tissue as input can be trained to predict outcome of patients with colorectal cancer (CRC). The tissue samples were obtained from the primary tumour using a tissue microarray (TMA) approach and stained for basic morphology using haematoxylin and eosin (H&E) only. The TMAs represent a comprehensive series of CRC patients with associated clinicopathological data and known disease-specific outcome. We developed and trained a machine learning model to directly predict the five-year disease-specific outcome with only one TMA spot image per patient as input. In this setting, the model solves a binary classification task and outputs a probability of survival at five years after primary CRC diagnosis. We also assigned the same task to three experienced pathologists from two different institutions, asking them to estimate the likelihood of survival at five years after the CRC diagnosis based on same set of TMA spot images. A majority vote of the three pathologists formed the Visual Risk Score. We compared the performance of the automated analysis against that of the pathologists and observed that the machine learning based approach outperformed the human observers in stratification of patients into low-risk and high-risk categories. The machine learning-based method also outperformed histological grade, assessed based on conventional microscopy analysis of the whole-slide tumour sample. We observed that our digital risk score is independent of both histological grade and stage of disease in a multivariate survival model. This observation implies that even a small tissue section of a TMA spot contains valuable information about morphological properties of the tumour and the disease outcome. To build a clinically useful prognostic classifier, the suggested model should be trained on whole-slide samples and evaluated on an extended patient series including data from different hospitals and diagnostic laboratories.

In our image-processing pipeline, we applied two deep learning methods i.e. convolutional and recurrent neural networks and combined these techniques to propose a model that is scalable to images of different size and could serve in assessment of CRC tumour samples in a digital pathology workflow. Particularly, we applied transfer learning by utilizing a ready-to-use visual recognition model (VGG-16) to avoid training a convolutional neural network from scratch. We show that a deep convolutional neural network trained on a large-scale dataset from a different domain is also useful for pattern recognition in digitized images of CRC. An alternative approach would be to learn unsupervised features on the dataset with, for example a convolutional autoencoder and then train a classifier. This would be more time-consuming as compared to taking an off-the-shelf feature extractor.

As with any other convolutional neural network, the VGG-16 model is bound to a fixed input image size and does not scale to large images. We addressed this problem with a recurrent neural network - an LSTM model - that we trained to “read” the input images piece by piece. Due to their recurrent nature, LSTMs can process arbitrary sequences of inputs and memorize long-term dependencies. Thus, a risk score could be evaluated on a tissue section of arbitrary size. Improvements on the proposed neural network setup would include employing a loss function tailored for time-to-event data to particularly eliminate the problem of unbalanced classes and reach better accuracy. The prognostic accuracy of a deep learning model that takes a whole-slide image as input also remains to be established. Even though we have applied various regularization techniques, including early stopping and dropout to prevent overfitting, additional validation on extended datasets is required to assess the generalizability of our findings.

In the context of diagnostic decision-making, however, it is necessary to know what are the factors affecting the final decision of the classifier. Dissecting knowledge hidden in an artificial neural network is arguably a challenging task. We made an effort to interpret LSTM activations with the intention to assess whether the network had learnt to detect tissue patterns representing known morphological entities. To our knowledge, only a few attempts have been made so far to interpret the internal behaviour of LSTMs^40,41^ and none in the field of tissue image analysis. Even though we identified that some units of the LSTM architecture have learnt to recognise biologically relevant tissue patterns, we have essentially only limited understanding of what drives the LSTM predictions and this analysis therefore needs to be considered exploratory. Overall, evaluation of the prognostic performance of different machine learning models suggested that deep learning techniques enable a more accurate outcome prediction as compared to an experienced human observer.

Taken together, we demonstrated that a machine learning based approach can outperform a visual prognostic assessment based on TMA spots as well as based on histological grading performed on whole slide tissue sections. The suggested method for analysis of CRC tissue samples stained for basic morphology only, could serve as a digital prognostic biomarker and further studies are warranted to evaluate the prognostic performance of the method in other patient series and with larger tumour tissue areas as input.

## Methods

### Patients and samples

The dataset consists of a series of 641 consecutive patients diagnosed with colorectal cancer and who underwent primary surgery at the Helsinki University Central Hospital in 1989–1998^33^. Ethical clearance was approved by the local operative ethics committee of The Hospital District of Helsinki and Uusimaa (Dnro HUS 226/E6/06, extension TMK02 §66 17.4.2013). Also, clearance from the National Supervisory Authority for Welfare and Health (Valvira) for using human tissues has been approved (Dnro 10041/06.01.03.01/2012). According to the Ministry of Social Affairs and Health, Finland Act on the Medical Use of Human Organs, Tissues and Cells, written informed consent was not required because patient consent could not be obtained from the persons since the study was retrospective and the amount of specimen was extensive. Tissue slides were scanned by a unique study ID and without patient identifiable data and linked to patient ID. All experiments were performed in accordance with relevant guidelines, and protocols were approved by the Research Director of the Institute for Molecular Medicine, Finland.

Out of 641 patients, 15 patients were excluded due to postoperative death or misdiagnosis. The patients represent a consecutive series and thereby it includes a variation of different grades and stages. For 23 patients, the TMA spot images were not representative or contained no tumour and these patients were also excluded. Training of machine learning algorithms such as deep neural networks generally benefit from a balanced dataset^42^. Therefore, 182 survivors were randomly removed to achieve equal numbers of patients who survived and died of colorectal cancer within five years after diagnosis, that is 420 patients.

For the construction of tumour tissue microarrays (TMAs), the original primary tumour tissue blocks were sampled with a 1.0 mm core needle using semiautomatic tissue microarray instrument (Beecher Instruments Inc., Silver Spring, MD, USA). Three cores per patient were punched from the paraffin-embedded tumour blocks and assembled into TMA blocks. The construction of the TMAs was blinded to patient outcome and the tissue cores were chosen so that they consist of the most representative, typically the least differentiated part of tumour. Four-micrometre thick sections were cut from the TMA blocks, stained with haematoxylin and eosin (H&E) and digitized with a whole-slide scanner (Pannoramic 250 FLASH, 3DHISTECH Ltd., Budapest, Hungary) with the image resolution of 0.22 μm/pixel. The TMA spots were annotated, linked to patient ID and extracted from the whole-slide TMA images using whole-slide image management software (WebMicroscope, Fimmic Oy, Helsinki). The extracted TMA spot images were cropped from the center of the image (3500 × 3500 pixels) to remove surrounding white background.

Follow-up time and outcome information is available for each of the patients as well as clinicopathological characteristics of the tumour samples including established predictors such as Dukes’ stage and histological grade. Histological characterization of the original whole-slide tumour samples was performed according to the WHO criteria and clinical, and outcome data was obtained from hospital, and pathology reports and the Finnish Population and Register Center and Statistics Finland. In addition to the histological grade assessed at the time of primary diagnosis, an individual risk score was assigned to each patient based on visual assessment of each of the TMA spots by three experienced pathologists (SN, CV and PK). The pathologists were asked, without knowledge of patient outcome, to classify the patients into long-term and short-term survivors. A majority rule was used to combine the individual assessments by the pathologists into a joint score, assigning a patient to the high-risk group when at least two experts marked the patient to be at high risk. We refer to this joint score as the Visual Risk Score. The Visual Risk Score was assessed only on a TMA level allowing direct comparison of the results between the human observer and the automated analysis. Thus, the Visual Risk Score captures features related to the level of differentiation of the tumour, but also other known prognostically relevant tissue characteristics.

### Feature extraction

Since the input TMA images were large (3500px x 3500px; 12 megapixel), the analysis started by splitting each image into tiles of 224 by 224 pixels. Tile size is determined by the convolutional neural network that we used as a feature extractor. The transfer learning^36,37^ approach we applied uses the intermediate activations of the VGG-16^34^ convolutional neural network from the second last fully-connected layer. The VGG-16 network is currently one of the most popular models in computer vision. This network has been trained on the ImageNet dataset^35^ and we took the model off-the-shelf without additional fine-tuning. VGG-16 activations were extracted from each tile individually. In total 256 tiles (16 rows and 16 columns) were generated from each input image so that the neighbouring tiles overlapped by 15 pixels. Each of these tiles were then passed through the VGG-16 pre-trained convolutional neural network to obtain a 4096-bin feature vector by saving activations of the second last fully-connected layer. Tile images were first normalized by subtracting a fixed value from each colour channel (Red: 123.68; Green: 116.779; Blue: 103.939) according to the mean pixel values computed on the VGG-16 training data from ImageNet.

### Classification and Training

For classification, i.e. five-year disease-specific outcome prediction, we used a three-layer 1D LSTM network. The network consisted of 264 cells at the first hidden layer, 128 cells at the second layer and 64 at the third layer. We used hyperbolic tangent as activation function in all hidden units A binary cross-entropy loss function and an adaptive learning rate method for gradient descent, Adadelta^43^ with default parameters (learning rate 1.0, decay 0.0) were used to train the LSTM. The LSTM model received a sequence of 4096-bin feature vectors extracted from individual tiles of TMA spot images. This approach is similar to purely recurrent ReNet architecture^44^ that swipes over a sequence of consecutive image tiles. Instead of using densely-connected LSTM cells we employed conventional way of extracting features form image data through a sequence of convolutional and pooling layers. Thus, the LSTM model performed feature extraction, image summarization and classification jointly as one task. Glorot uniform initializer^45^ was used for kernel weights initialization and orthogonal initializer for recurrent weights of LSTM. The experiments were performed in three-fold cross-validation. We used 220 samples for training the LSTM model, 60 for validation and 140 for testing at each fold. Since modern neural network architectures are typically over-parametrised and are prone to overfitting, we applied different regularization techniques: Elastic Net regularization (l1: 0.005; l2: 0.005) was used at each hidden layer of the LSTM and a Dropout of 5% was applied to the input layer and to the last hidden layer of LSTM. In addition, early stopping was used for each fold to prevent overfitting.

In addition to the LSTM method we used traditional machine learning classifiers including Logistic Regression, Naïve Bayes and Support Vector Machine. We did not attempt to determine the best possible settings for the classifiers. Instead, our aim was to compare the performance of the proposed deep architecture to the baseline performance achieved by the traditional classifiers, whereas rigorous comparison of the classifiers was out of the scope of this study. Scikit-learn^46^ (version 0.18.1) implementation of the classifiers was used with default parameter setting. Logistic regression was trained with L2 regularization (1.0) and ‘liblinear’ optimizer. Since input features are continuous normally-distributed values we, used Gaussian Naïve Bayes. Finally, the Support Vector Machine was used with a linear kernel and penalty parameter C set to 1.0. Tolerance for stopping criterion was set to 10^−3^. Since these classification algorithms are well-studied and are known to perform well in most circumstances, their performance was used as a reference to the LSTM classifier.

In contrast to the LSTM, the rest of the algorithms do not scale to images of arbitrary size and require additional steps to pull together features extracted from individual tiles and produce a global descriptor of a TMA spot. To this end, we used Improved Fisher Vectors^47^ (IFV) encoding with 32 mixture components, compressing the local descriptors into 32 components with principal component analysis. IFV is an orderless pooling encoder that describes a set of local descriptors with a single vector based on their first and second order statistics. Finally, the 2048-dimensional IFV representation of the image served as input to the traditional classifiers.

We used area under the ROC curve to compare the accuracy of the classifiers. To compare the difference between two ROC curves we applied Venkatraman^48^ permutation test.

### Survival analysis

Time to event was time from diagnosis to death of colorectal cancer. Patients alive at the end of follow-up or who died of causes unrelated to colorectal cancer were censored. We used the Kaplan-Meier method to estimate survival curves^49^ and statistical significance of the difference observed between patient groups was evaluated with the log-rank test. For survival analysis and to simplify the interpretation of the results, the patient series was split into low- and high-risk groups at the median value of the risk score generated by the LSTM model in each fold of the test set. Cox proportional hazards model^50^ was used to estimate effect sizes (hazard ratios) of the covariates individually (univariate) and adjusted for the effect of other covariates (multivariate).

### Implementation

We used Keras^51^ - a python framework for deep learning to implement feature extraction with VGG-16 network and to train the LSTM model for outcome prediction. Training was performed on NVIDIA Tesla k40 graphical processing units (GPUs) at the Center for Scientific Computing (CSC IT Center for Science, Espoo, Finland). Survival analysis was implemented in an R statistical environment with “survival” package available from CRAN^52^.

## Electronic supplementary material

Supplementary Information
