# Supplementary material for: Deep learning based tissue analysis predicts outcome in colorectal cancer
Source: Sci Rep. 2018 Feb 21;8:3395. doi: 10.1038/s41598-018-21758-3 (PMC5821847; doi:10.1038/s41598-018-21758-3)
Supplement: Supplementary file 1 — Supplementary Information [file 41598_2018_21758_MOESM1_ESM.pdf]

Deep learning based tissue analysis predicts outcome in colorectal cancer

Dmitrii Bychkov[a]*, Nina Linder[a,b]**, Riku Turkki[a]**, Stig Nordling[c], Panu E. Kovanen[d], Clare Verrill[e], Margarita Walliander[a], Mikael Lundin[a], Caj Haglund[f,g], and Johan Lundin[a,h].

* corresponding author (dmitrii.bychkov@helsinki.fi)

** these authors contributed equally to this work

[a]Institute for Molecular Medicine Finland, University of Helsinki, Helsinki, Finland; [b]Department of Women's and Children's Health, International Maternal and Child health (IMCH), Uppsala University, Sweden; [c]Department of Pathology, Medicum, University of Helsinki, Helsinki, Finland; [d]Department of Pathology, University of Helsinki, and HUSLAB, Helsinki University Hospital, Helsinki, Finland; [e]Nuffield Department of Surgical Sciences, University of Oxford, UK and the NIHR Oxford Biomedical Research Centre; [f]Department of Surgery, University of Helsinki and Helsinki University Hospital, Helsinki, Finland; [g]Research Programs Unit, Translational Cancer Biology, University of Helsinki, Helsinki, Finland; [h]Department of Public Health Sciences, Global Health/IHCAR, Karolinska Institutet, Stockholm, Sweden;

SUPPLEMENTARY DATA

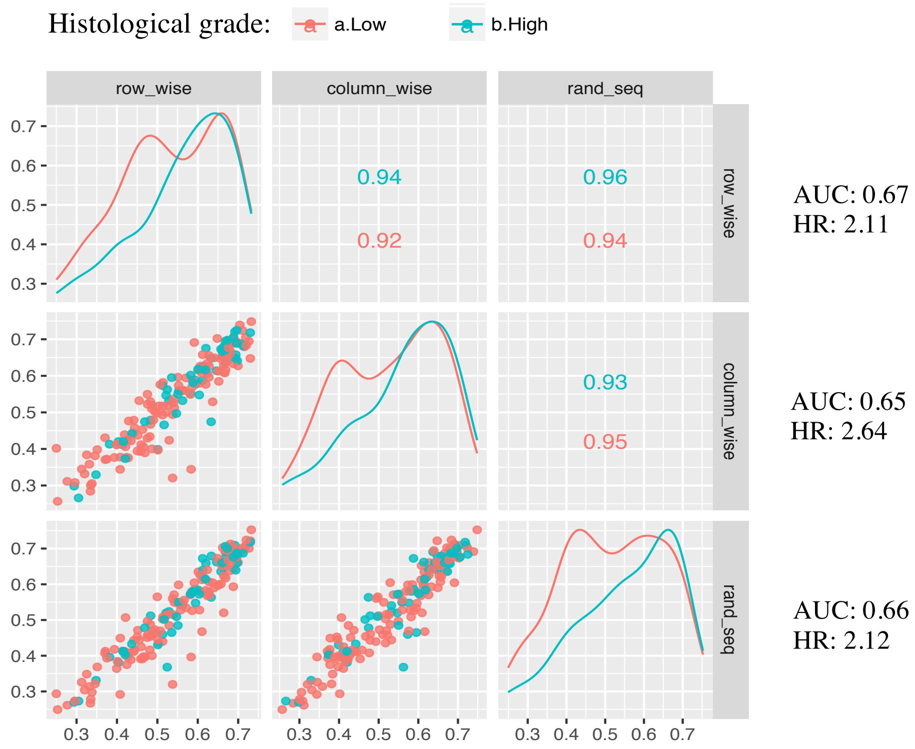

Figure 1 – Model performance on different tile orderings for 1D-LSTM. We tested row-wise, column-wise as well as random tile ordering within individual TMA spots. The pairplot suggest that predictions are highly correlated. This means that on our dataset the order in which tiles are fed to the LSTM is not significant. AUCs and Hazard rations were also on the same level.

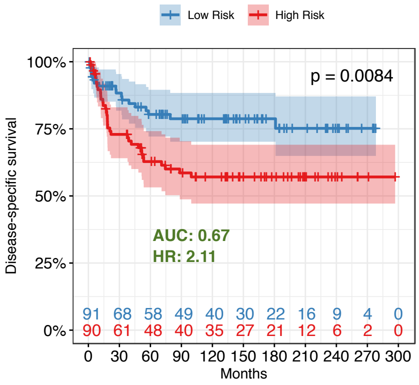

Figure 2 – Performance of the deep learning model on held out 181 patients not used in cross-validation. As most of the cases (128/181) are survivors, Kaplan-Meier curves appear high on the plot.

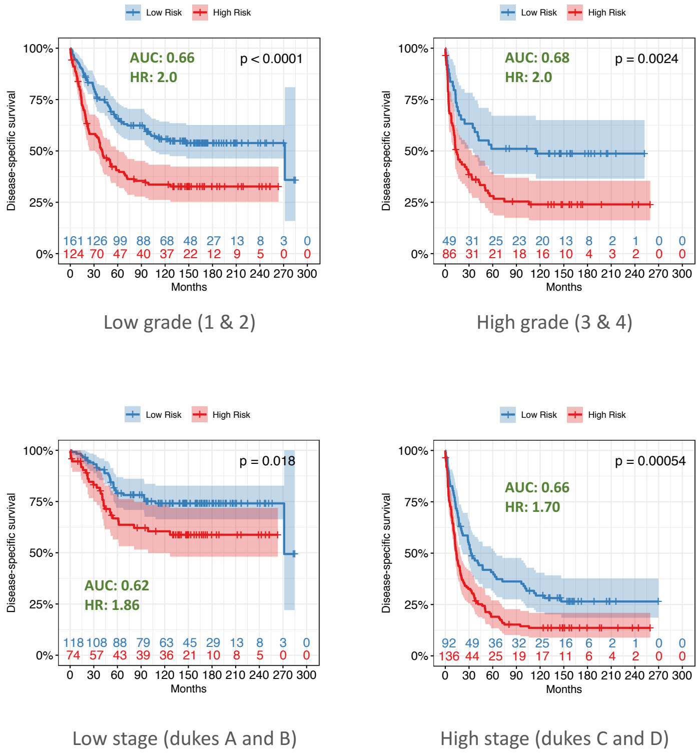

Figure 3 – Performance of the deep learning model based on different clinicopathological characteristics: Histological grade (upper row) and Dukes' stage (bottom row). We can see that predictions are consistent and that model performance is approximately the same across different patient subgroups.
